# Menaquinone biosynthesis inhibition: a review of advancements toward a new antibiotic mechanism

**DOI:** 10.1039/c7ra12950e

**Published:** 2018-01-29

**Authors:** M. Boersch, S. Rudrawar, G. Grant, M. Zunk

**Affiliations:** School of Pharmacy and Pharmacology, Griffith University Gold Coast Queensland 4222 Australia m.zunk@griffith.edu.au; Quality Use of Medicines Network, Griffith University Gold Coast Queensland 4222 Australia; Menzies Health Institute Queensland, Griffith University Gold Coast QLD 4222 Australia

## Abstract

Menaquinone is essential in electron transport and ATP generation in all Gram-positive, and anaerobically respiring Gram-negative bacteria. By inhibiting menaquinone production in target organisms, bactericidal action can be achieved irrespective of the organisms' growth phase. This pathway is absent in human cells, as menaquinone is obtained only through the diet. This paper provides a succinct review of major advancements, where present, at all enzymatic steps of the biosynthetic pathway of menaquinone. Structure–activity relationships are evaluated, as well as results translating these relationships to growth inhibition studies.

## Abstract 2

Menaquinone is essential in electron transport and ATP generation in all Gram-positive, and anaerobically respiring Gram-negative bacteria. Inhibition of menaquinone production at different steps of the biosynthesis pathway has shown promising novel antibacterial action.

## Graphical abstract

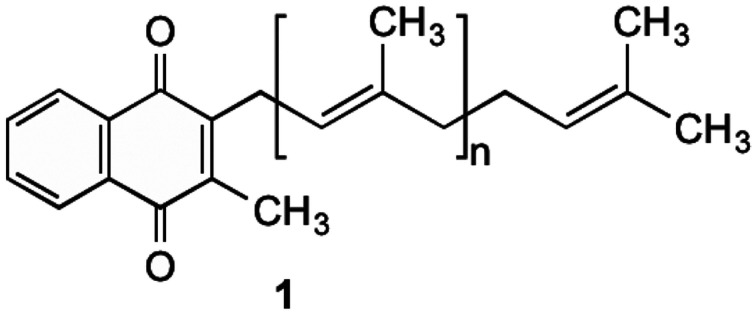

## Menaquinone

**Fig. 1:**
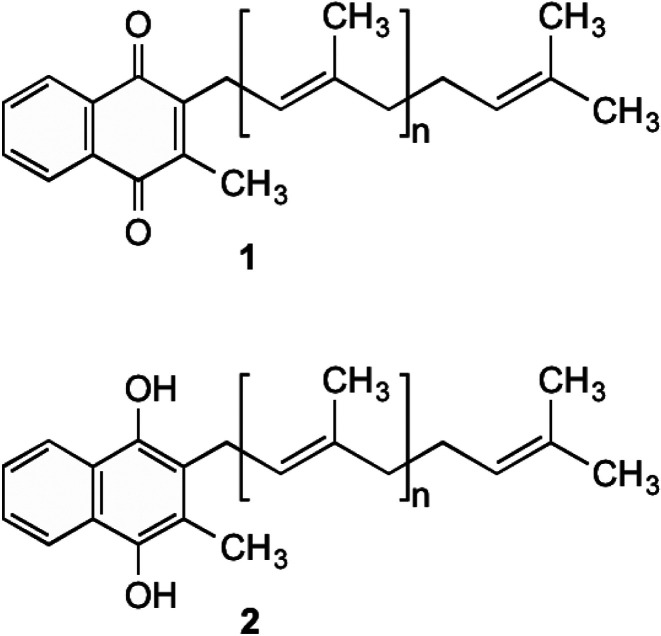
MK (1) and MKH_2_ (2).

Menaquinone (MK, 1) (Fig. 1) plays an important role in electron transport in all Gram-positive, and anaerobically respiring Gram-negative bacteria.^1–6^ It is the primary form of electron transport between NADH dehydrogenase II, succinate dehydrogenase, cytochrome bc_1_ complex, as well as nitrate and fumarate reductase enzymes, which are present in the bacterial cell membrane. In these steps, MK (1) is reduced by two electrons to produce menaquinol (MKH_2_ (2)) and shuttles these electrons to an acceptor in the next step of the chain.^7^ This substrate oxidation process provides the energy needed to maintain the proton gradient and potential energy used by the F_0_F_1_ATPase complex to convert ADP into ATP. This is done through a proton ion channel that allows the protons to flow down the gradient, which causes a rotational conformational change in the complex resulting in the movements that produce and release ATP.^8^ Without this shuttling of protons the gradient would collapse and ATP synthesis would cease. Unlike bacteria, humans utilise ubiquinone within their electron transport chain in order to shuttle electrons. Therefore MK (1) biosynthesis is absent, with 1 being obtained through dietary sources.^9^

Although MK's (1) role in humans is not completely understood, it is hypothesised that 1 is utilised to produce clotting factors if phylloquinone (vitamin K_1_) is unavailable.^10–12^

## Bacterial synthesis of menaquinone

**Scheme 1:**
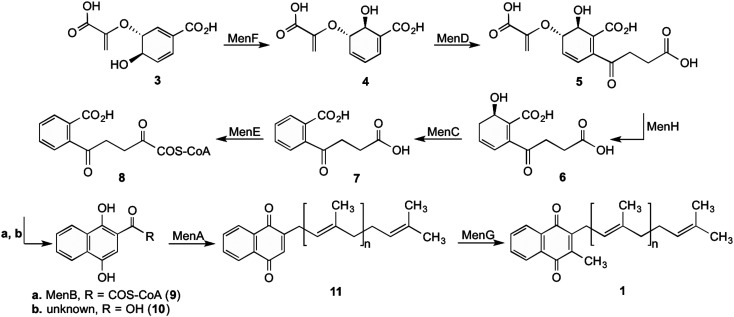
Biosynthetic pathway for 1 in *E. coli*.^1^

The synthesis of 1 by bacteria is a seven-step process (Scheme 1) which starts with chorismate (3), a branch-point intermediate supplied by the shikimate pathway.^1,2,6,13–15^ Isochorismate synthase (MenF) converts 3 into isochorismate (4), which in turn is converted to 2-succinyl-5-enolpyruvyl-6-hydroxy-3-cyclohexene-1-carboxylate (SEPHCHC, 5) by MenD.^16^ The aromatic intermediate OSB (7) is produced in a two-step process involving MenH and MenC *via* the intermediate compound SHCHC (6). The carboxyl group in 7 is then activated by MenE through an ATP dependent process to produce *O*-succinylbenzyl-CoA (OSB-CoA, 8), which allows MenB to catalyse the cyclization of this product forming the naphthalenoid skeleton DHNA-CoA (9). This skeleton is then hydrolysed through an unknown Men enzyme creating DHNA (10) which is decarboxylated and prenylated by MenA to produce DMK (11).^17^ The final step in the pathway involves MenG adding a methyl group to position 3 of the naphthalenoid ring of 11 creating MK (1).

All enzymes of this pathway are enticing targets for inhibition as they are extremely conserved in all Gram-positive and facultative anaerobic Gram-negative bacteria.^16^ Interestingly, not all of the enzymes have had significant investigation into potential inhibitors.^18–26^ To date the most promising results have come from inhibitors targeting the enzymes further downstream of the synthesis pathway. Major inhibitors that have been discovered to date will be reviewed in this article.

### MenD: 2-succinyl-5-enolpyruvyl-6-hydroxy-3-cyclohexene-1-carboxylate synthase

MenD, the second enzyme involved in the biosynthesis of 1 is a thiamin diphosphate-dependent decarboxylase that catalyses the creation of SEPHCHC (5) by a ping-pong mechanism involving α-ketoglutarate.^1,14^ Investigations into inhibitions of this enzyme have been conducted by Fang *et al.*^13^ by using acylphosphonate derivatives as false substrates of α-ketoglutarate. Of the eleven compounds that they synthesised, two compounds were effective inhibitors.

**Fig. 2:**
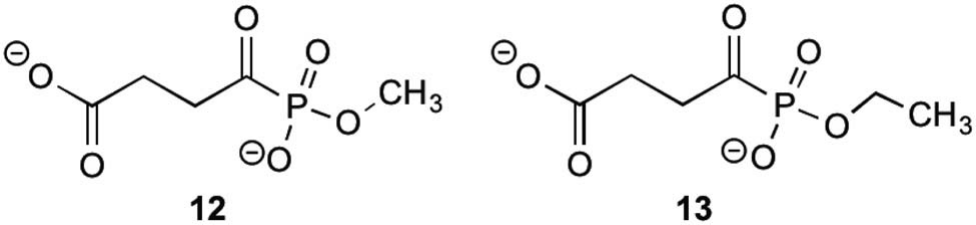
The acylphosphonates 12 and 13.^13^

These two compounds (Fig. 2), methyl succinylphosponate (12) and ethyl succinophosphonate (13), showed enzyme inhibition (*K*_i_) at 0.7 μM and 16 μM concentrations, respectively. It was observed that although a great improvement in the inhibitory action is achieved by addition of an alkyl group to the phosphonate, when larger alkyl groups were substituted at this position less inhibitory action was produced. Similarly, it was shown that esterification of the carboxylate group by alkyl addition rendered the inhibitor ineffective. Whilst investigating additions to this ester group, a phenyl ester showed improved inhibitory action when compared to its alkyl ester counterparts. This was hypothesised by Fang *et al.*^13^ to be due to potential π-stacking with amino acid residues in the active site corresponding to the aromatic groups position, however with a *K*_i_ value of 160 μM, this is much less effective than simply keeping the carboxylate group present.

Further work into inhibition of MenD has also been carried out by Xu,^27^ who managed to synthesise thiamine diphosphate analogues with activity against MenD. Four analogues were successfully synthesised showing moderate enzyme inhibition, however when it came to growth inhibition the results were less promising. Only one analogue showed growth inhibition at concentrations <200 μM, with the others inhibiting growth at concentrations 3–10 times more than this. It can be concluded that these analogues require further investigation to translate their moderate enzyme inhibition to growth inhibition. Specific structures of these thiamine diphosphate analogues are available in Xu's publication.^27^

### MenE: *O*-succinylbenzoate synthase

The fifth enzyme in the bacterial synthesis of 1, MenE catalyses the addition of S-CoA to OSB *via* an OSB-AMP intermediate.^15,23,25,28–30^ After initial location and characterization of the MenE gene by Sharma,^29^ varying approaches have been undertaken to attempt to inhibit this enzyme. The reaction mechanisms of MenE have been proposed by Tian *et al.*^15^ where they describe the reaction as a Bi Uni Uni Bi ping-pong mechanism in the presence of ATP, *via* an acyl-AMP intermediate.^15^ The adenylation of OSB by ATP creates the OSB-AMP intermediate, before ligation of CoA *via* thioesterification produces OSB-CoA.

**Fig. 3:**
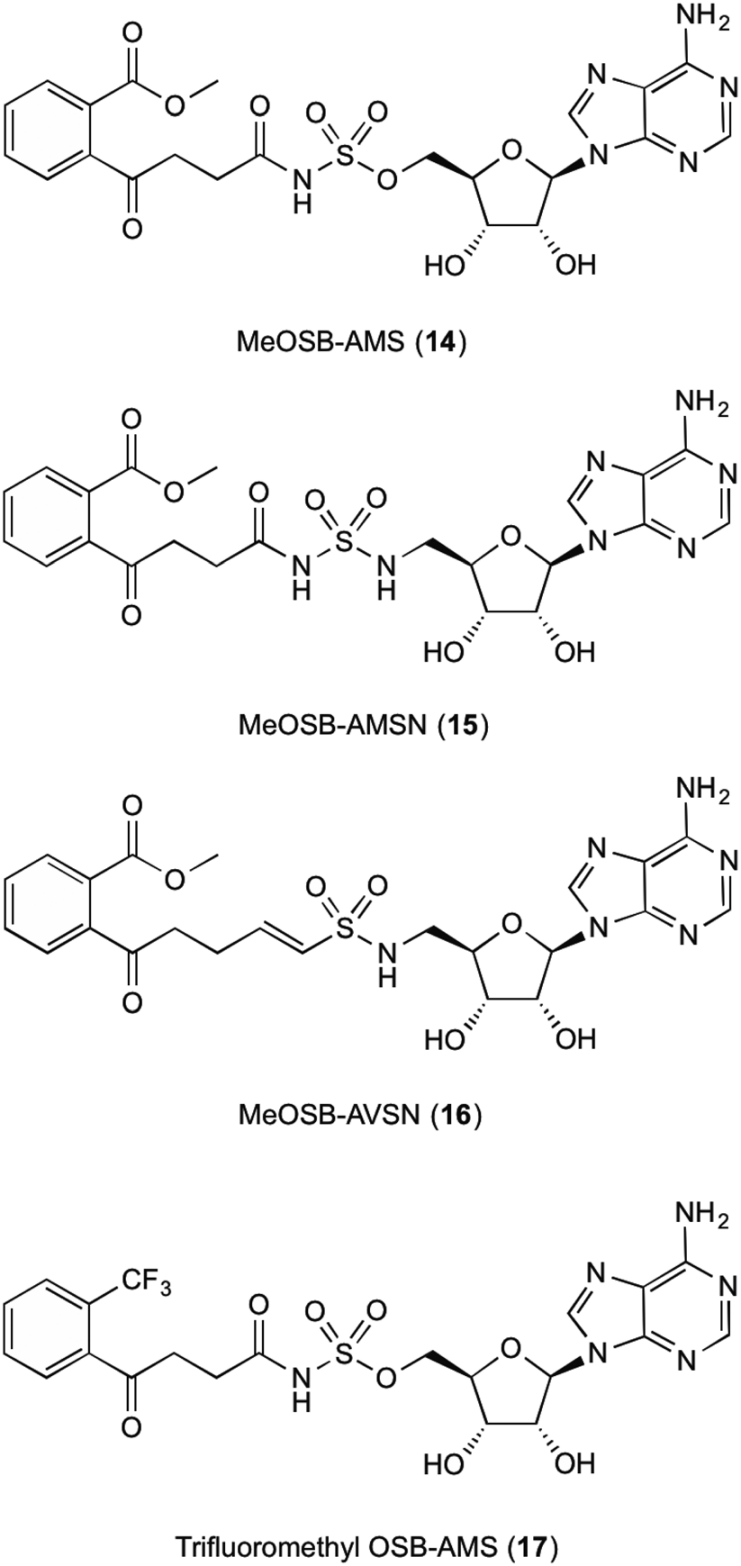
MenE inhibitors published by Lu *et al.*^15,23^

Efforts into creating an inhibitor for this enzyme have mostly been centred on creating variations on the OSB-AMP intermediate structure, likely due to the instability of OSB itself. Lu *et al.*^23^ initially created inhibitors by using this approach. Three inhibitors were synthesised which showed ability to inhibit MenE. In these created compounds, linkages mimicking the phosphate AMP linkage were replaced by bioisosteres sulphamate 14, sulphamide 15 and vinyl sulphonamide groups 16 (Fig. 3). Of these three, the vinyl sulphonamide variation provided an IC_50_ of only 5.7 μM.^30^ This is particularly interesting, as Lu *et al.*^30^ outlines, the vinyl sulphonamide will trap the incoming CoA thiol nucleophile, stopping the reaction from proceeding, instead of just mimicking the intermediate structure for active site competition.^30^

The mechanism of this nucleophile entrapment is explained clearly in their paper. Kinetic analysis performed by Lu *et al.* also showed that these compounds are slow binding inhibitors, which suggests a change in conformation during substrate binding.^30^ It was also discovered that the presence of the ketone on the OSB moiety is important, as the exo-methylene groups showed greatly reduced IC_50_ values. This might be due to the carbonyl becoming involved in essential hydrogen bonding interactions within the active site of the enzyme.^30^ Similar efforts from Tian *et al.*^15^ involved using a trifluoromethane substituted OSB 17, linked to AMP *via* a sulphonamide linkage. Here it was reported that this substitution provided a potent mixed inhibitor of the enzyme at both the ATP and OSB binding sites. Although this was potent, the trifluoromethane substitution did not create an inhibitor that was as active as compounds containing the original carboxylate functional group. The activity of this inhibitor, however, was not translated in microbial growth studies, as it was assumed that the lack of growth inhibition was due to poor penetration into the cell. Further explanations into reasons why were not provided or investigated by Tian *et al.*^15^ Building on from this work, Lu *et al.*^23^ then investigated the efficacy of varying substitutions at the OSB carboxylate group. By introducing a methyl ester to OSB, authors found that although IC_50_ values were at best 14 μM, these were also not as potent as the original carboxylic acid moieties^23^ which is in line with the findings of Tian *et al.*^15^

### MenB: 1,4-dihydroxy-2-naphthoyl-CoA synthase

1,4-Dihydroxy-2-naphthoyl-CoA synthase is the 6^th^ enzyme involved in the production of MK (1) in bacteria, catalysing the creation of DHNA-CoA (9) from *O*-succinylbenzoate-CoA *via* a Claisen condensation. The structure of MenB was first reported by Truglio *et al.*^32^ in 2003 after a crystal structure of this enzyme was obtained from enzyme extracted from *M. tuberculosis*. The crystals were able to be soaked in acetoacetyl-CoA and an X-ray diffraction pattern produced. This not only allowed structure determination of the quaternary structure, but also allowed the active site to be catagorised and a catalytic mechanism proposed. Obtaining crystals of natural substrate bound into the active sight was not able to be achieved as the substrate is too unstable to exist for long periods of time, reacting with itself to form spironolactone moieties. From the obtained enzymatic structures, it was determined that MenB is a member of a superfamily of enzymes called the crotonase superfamily.^32,33^

Initial investigations into potential inhibitors of this enzyme were conducted by Li *et al.*^34^ where a coupled assay was used to screen over 105 000 drug-like molecules with a large structural diversity. From this bank of molecules, 455 molecules showed at least 30% enzyme inhibition relative to control, of which four compounds with a benzoxazine scaffold were chosen for further investigation. Using this scaffold structure as a theme, 13 compounds (18a–m) were synthesised to attempt to increase inhibitory action (Table 1).

**Table 1:** Compounds synthesised by Li *et al.*^34^ MIC values obtained using *M. tuberculosis*

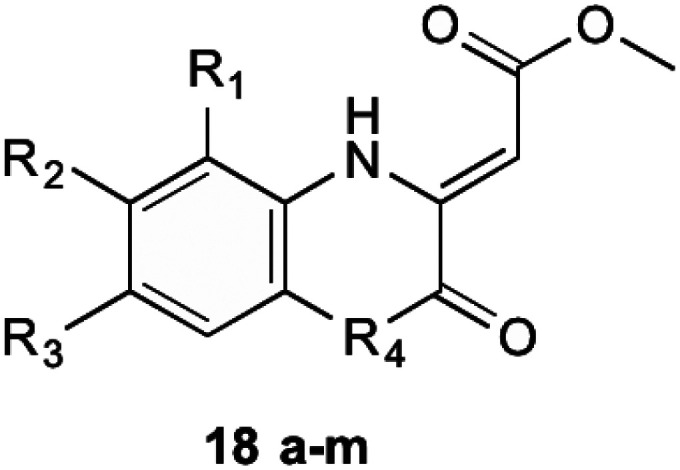	
Compound 18	MIC (μg/mL)
(a) R_1_ = H, R_2_ = H, R_3_ = H, R_4_ = O	0.64
(b) R_1_ = Me, R_2_ = H, R_3_ = H, R_4_ = O	25
(c) R_1_ = H, R_2_ = Me, R_3_ = H, R_4_ = O	>100
(d) R_1_ = H, R_2_ = F, R_3_ = H, R_4_ = O	0.63
(e) R_1_ = H, R_2_ = Cl, R_3_ = H, R_4_ = O	5
(f) R_1_ = H, R_2_ = NO_2_, R_3_ = H, R_4_ = O	50
(g) R_1_ = H, R_2_ = EtSO_2_, R_3_ = H, R_4_ = O	>100
(h) R_1_ = H, R_2_ = H, R_3_ = Me, R_4_ = O	100
(i) R_1_ = H, R_2_ = H, R_3_ = F, R_4_ = O	0.63
(j) R_1_ = H, R_2_ = H, R_3_ = Cl, R_4_ = O	0.63
(k) R_1_ = H, R_2_ = H, R_3_ = NO_2_, R_4_ = O	>100
(l) R_1_ = H, R_2_ = H, R_3_ = H, R_4_ = NH	>100
(m) R_1_ = H, R_2_ = H, R_3_ = H, R_4_ = S	>100

Through these investigations important structural activity relationships were uncovered by Li *et al.*^34^ Firstly, growth inhibition was generally increased when substituting a halogen upon the benzene ring. Conversely, a reduction in growth inhibition was seen when bulky alkyl groups were substituted upon the benzene ring structure, regardless of their electron withdrawing or donating nature. This shows that steric interactions at this section of the molecule are important for bactericidal action. Side chain size was also shown to be important. When the side chain methyl ester was substituted with a chlorophenone group, a great reduction in bacterial growth was observed. It was investigated, however, that even though the chlorophenone substituted compounds achieved a reduction in bacterial growth, no direct inhibition of MenB was seen from enzymatic inhibition studies. This suggests that these compounds might have multiple targets within the cell. Furthermore, Li *et al.*^34^ showed that when using a different core ring structure from the benzoxazine, the ability to impede bacterial growth was substantially diminished. This was shown by creating compounds with a quinoxaline or benzothiozine core structure.

Further to these results from the same screening assay, Li *et al.*^34^ identified 7 compounds with a backbone of 2-aminobutanoates, a structure similar to the natural substrate OSB. Although these compounds were initially theorised to inhibit either MenB or MenE, there was no reported inhibition of MenE. These compounds were also found to demonstrate poor correlation between enzyme inhibition and antibacterial activity. This was theorised to be due to a retro-Michael addition reaction of the substrates, degrading their structure. This was important in the results as the half-life of the created structures was found to be approximately 10 minutes, and the MIC assays take 24–48 hours to complete.

To circumvent this, compounds unable to undergo retro-Michael addition were created. It was found that electron withdrawing groups substituted on the aromatic ring trended to show a higher antibacterial action, particularly at the 2 and 4 position, which is the same theme found in their earlier research on inhibitors mentioned previously. The most successful of these compounds are shown in Table 2 (19a–e). These compounds were then further tested by Matarlo *et al.*^21^ and shown that the MIC values for the methyl butanoate are as low as 0.35–0.75 μg mL^−1^. Interestingly, Matarlo found that these compounds exert their effect after conversion to a CoA adduct *in vivo*, before binding to inhibit 1 synthesis.

**Table 2:** Compounds devised by Matarlo *et al.*^21^ which do not undergo retro-Michael addition

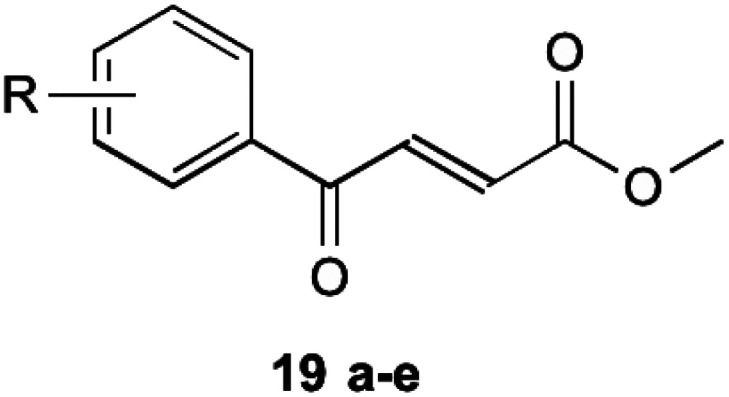			
Compound 19	R	MIC MSSA (μg mL^−1^)	MIC MRSA (μg mL^−1^)
a	4-Cl	0.35	0.75
b	4-F	8	12
c	4-Br	8	16
d	4-NO_2_	4	4
e	2,4-Cl	1	2

### MenA: 1,4-dihydroxy-2-naphthoate prenyltransferase

MenA is involved in the penultimate step in the synthesis of MK (1). It has been proposed that this enzyme has structural and functional similarities in its catalytic mechanism to the UbiA enzyme, a member of a class and family very similar to the MenA enzyme, which was deduced from amino acid sequence analysis.^35,36^ From the findings of these comparisons, it is shown that the active site of both enzymes are extremely similar, showing no activity in when key missense mutations are introduced to the same place. The proposed mechanism of action by Huang *et al.*^36^ shows a magnesium coordinated catalyst reaction where two magnesium atoms in the active site pocket are coordinated on either wall by amino acids D198 and D202 on the right, and D106 and N102 on the left. A highly conserved tyrosine motif around the active site entrance (Y139) is important in coordination of substrates towards this active site to allow the reaction to occur.^36^ This tyrosine motif is suspected to interact with the isoprenyl side chain to draw the chain into the active site to allow the catalytic reaction to occur, and as such lipophilicity at this site for any potential inhibitor is important. Coordination of DHNA (10) into the active site is primarily achieved through the presence of the carbonyl group. This carbonyl also plays a part in the proposed reaction mechanism, providing an environment which promotes electron movement into the ring structure of DHNA (10).

**Fig. 4:**
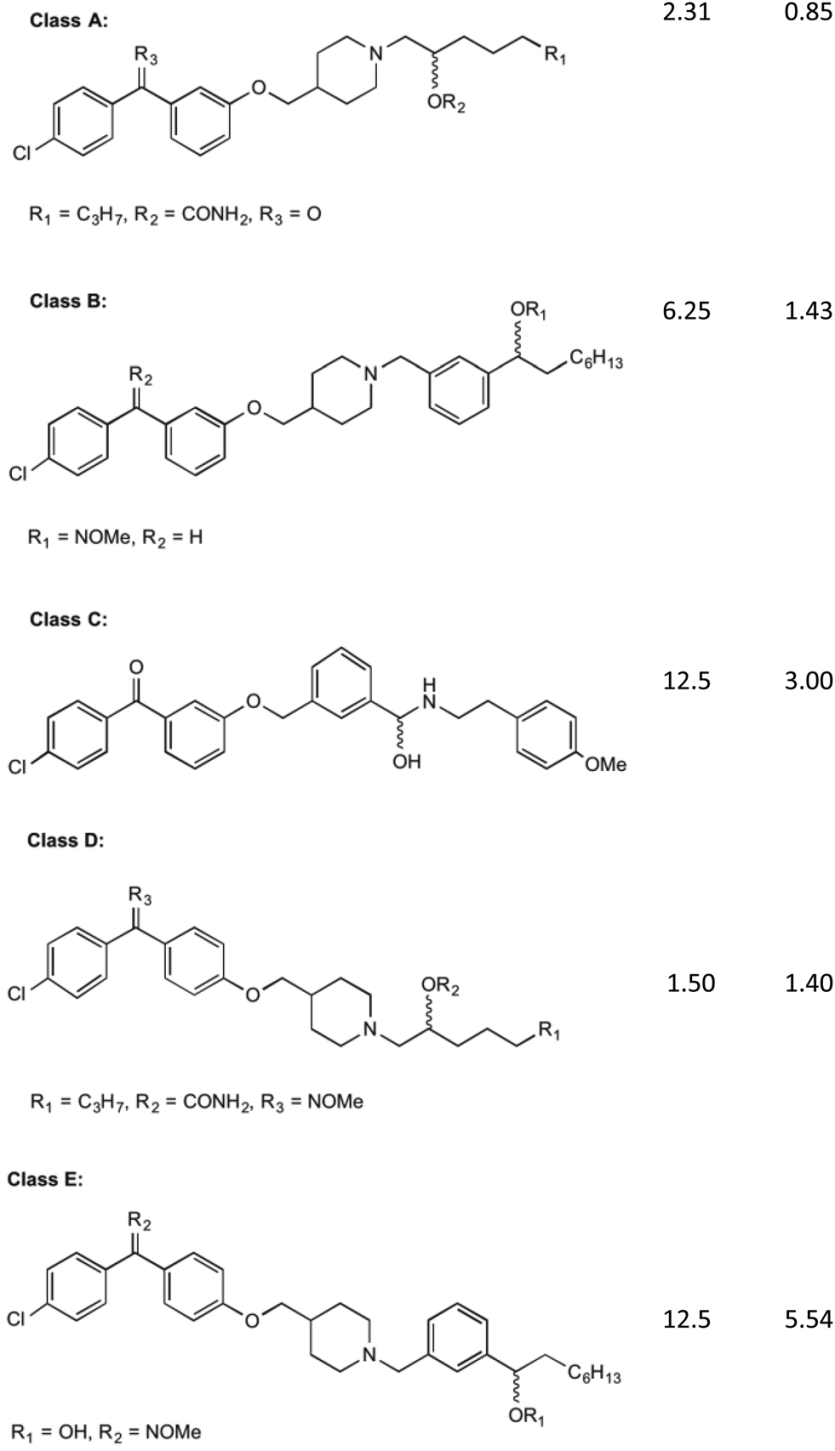
The five classes of compounds discovered by Debnath *et al.* with minimum inhibitory concentrations for both the microplate alamar blue assay, and the low-oxygen recovery assay using *M. tuberculosis*. The MIC values shown are of the best example discovered of each class.^37^

Initial investigations into the inhibition of this enzyme have yielded positive results. Most of this work has been pioneered by Kurosu *et al.*^17^ and Debnath *et al.*^37^ who have based much of their efforts on the creation of structures with similar SARs to aurachin RE, a natural product that shows bactericidal activity through a proposed dual mechanism of MenA and respiratory chain inhibition.^38^ Debnath *et al.*^37^ have been able to synthesise over 400 molecules of interest with >95% purity which have been screened in bacterial growth inhibitory assays, with varying results. The compounds synthesised have been based around a benzophenone core structure, with a highly varied lipophilic side chain substitution. It has been discovered by Debnath *et al.*^37^ that the position of a nitrogen atom in this tail is important to its bactericidal activity, with the most anti-mycobacterial activity achieved with a nitrogen 13 carbon-spaces away from the carbonyl on the benzophenone structure, yielding MIC of 0.85 mg L^−1^. Many variations of these compounds are displayed in their results tables, all catagorised into five broad classes (Classes A–E) of agents (Fig. 4).^39^

Of these classes, Class A showed to be the most effective, with the above-mentioned MIC values recorded from elements of this species of compound. The classes of compounds categorised differ in their variation in the backbone of the side chain and substitution of functional groups. It is also stated that the presence of a halogen on the first benzene ring improves bacterial killing potential, however the mechanisms for this have not been able to be explained clearly. These compounds were found to act *via* direct inhibition of the respiratory chain, as well as MenA inhibition. This was determined by Debnath *et al.*^37^ by running growth inhibitory assays under anaerobic conditions (determining MenA inhibition) and oxygen recovery assays against non-replicating *M. tuberculosis* (determining direct respiratory chain inhibition).^39^ Whilst the chirality of the variations on Class A inhibitors effected MIC values, chirality was not shown to have an effect on the activity of group B, C, D and E molecules.

It is worth noting the synthesis of all of these compounds involves costly catalysts and many steps. Although yields of each step have been acceptable (approx. 80% per step) and the products quite pure after workup, room for improvement can be made in reducing overall cost of manufacture of derivatives. Cytotoxicity was also investigated by Debnath *et al.*^37^ who found that initial effects on mammalian cell lines showed IC_50_ levels of 1–6.5 mg L^−1^. Through the use of an *O*-methyl oxime substitution this was reduced to an IC_50_ of 25 mg L^−1^. It is hypothesised that this substitution reduced the benzophenone electrophilicity allowing it to interfere less with the mammalian cell redox systems, although its specific mechanism is still largely unknown.^39^ Although this substitution reduced toxicity to mammalian cells, further decrease in toxicity is desired.

To date only work published from the laboratories of Debnath *et al.*^37^ has shown insight into the specific inhibition of MenA. It is also worth noting that extremely limited SAR studies have been characterised, as well as no literature showing the crystal structure of MenA is available. As such, active site interactions with substrates for this enzyme are not known with any clarity.

### MenG: demethylmenaquinone methyltransferase

MenG is the final step in the bacterial production of MK (1). It is responsible for the addition of the methyl group onto the aromatic ring of DMK (11). Although the structural similarities present with ubiquinone, MenG has no functional equivalence to ubiquinone methyltransferases. Work involving the discovery of therapeutic agents which inhibit MenG has been investigated by Benkovic *et al.*^40^ Here borinic ester derivatives were developed and found to have inhibitory action, and screened against known methyltransferase inhibitors sinefungin and S-adenosylhomocysteine (SAH). Whilst many derivatives were tested against *B. subtilis'* MenG methyltransferase, only one borinic ester 20 was found to have activity.

**Fig. 5:**
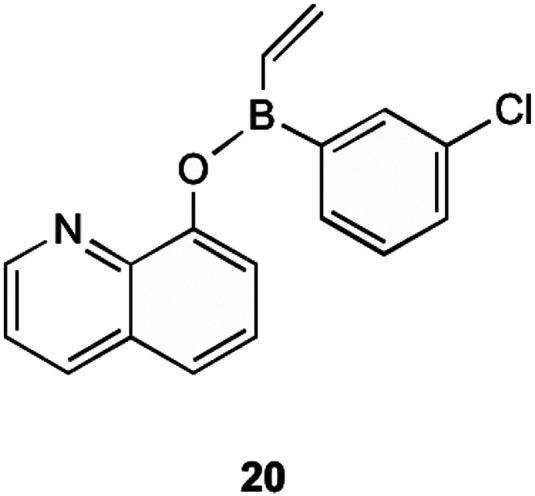
*meta*-Chloro borinic ester derivative 20 showing most promising MenG inhibition as investigated by Benkovic *et al.*^40^

As shown in Fig. 5, 20 is the *meta*-chloro derivative of the species of compounds. This showed activity comparable to sinefungin, the natural inhibitor already known. The results of their assays showed that at 100 μM concentration of this compound, only 40% of enzyme activity remained. When this chloro group was moved to a different position on the aromatic ring the activity was greatly limited, or in the case of removal, activity lost completely. It is worth noting that even with the chloro group present this structure was not as effective as SAH in inhibiting MenG, as inhibition with SAH resulted in <10% of enzyme activity remaining, but both SAH and sinefungin are not appropriate for therapeutic use due to their levels of toxicity to eukaryotic cells. Although the other borinic esters did not return as promising results with specific regards to MenG inhibition, a complete list of structures investigated can be found in their supplementary material, of which most had respectable MIC values against a range of bacterial species.^40^

In a unique approach, a direct MK (1) binding agent, lysocin E, was discovered by Hamamoto *et al.*^41^ Lysocin E is a cyclic peptide, that shows potent bactericidal activity against a variety of Gram-positive bacteria. This bactericidal activity results from bacterial membrane disruption, as menaquinone is present in small amounts in the bacterial cell membrane. Cytotoxicity studies were also conducted using mice models, and lysocin E was found to have low toxicity, with an acute toxic dose of >400 mg kg^−1^. Although this shows potential for therapeutic use, spontaneous mutants showing resistance to lysocin E were observed in a laboratory setting.^26,41^

## Conclusions

Great strides have been achieved in the progress towards inhibition of MK (1) biosynthesis in bacteria as a result of combined efforts of laboratories around the world. Advances in MenA inhibition provide the greatest developments into both potency and selectivity of many classes of novel antibacterial agents at this current stage. Although the efforts into MenA inhibition have proved fruitful, progress towards MenB and MenE inhibitors should also be followed closely as they too hold promising futures for research. As stated previously, this follows the observed trend of enzymes further down the synthesis pathway providing the most successful inhibitors. As such, more investigation into the earlier synthetic pathway enzymes would fill a current gap in the research.

## Conflicts of interest

The authors declare there are no conflicts of interest.

## Supplementary Material

## References

[cit1] Meganathan R. , Biosynthesis of the isoprenoid quinones menaquinone (vitamin K2) and ubiquinone (coenzyme Q), Escherichia coli and Salmonella: cellular and molecular biology, 1996, vol. 1, pp. 642–656

[cit2] Hiratsuka T., Furihata K., Ishikawa J., Yamashita H., Itoh N., Seto H. (2008). *et al.*, An alternative menaquinone biosynthetic pathway operating in microorganisms. Science.

[cit3] Dairi T. (2009). An alternative menaquinone biosynthetic pathway operating in microorganisms: an attractive target for drug discovery to pathogenic Helicobacter and Chlamydia strains. J. Antibiot..

[cit4] Collins M., Goodfellow M., Minnikin D. (1979). Isoprenoid quinones in the classification of coryneform and related bacteria. Microbiology.

[cit5] Meganathan R. (2001). Biosynthesis of menaquinone (vitamin K2) and ubiquinone (coenzyme Q): a perspective on enzymatic mechanisms. Vitam. Horm..

[cit6] Bentley R., Meganathan R. (1982). Biosynthesis of vitamin K (menaquinone) in bacteria. Microbiol. Rev..

[cit7] Kurosu M., Begari E. (2010). Vitamin K2 in electron transport system: are enzymes involved in vitamin K2 biosynthesis promising drug targets?. Molecules.

[cit8] Capaldi R. A., Aggeler R. (2002). Mechanism of the F(1)F(0)-type ATP synthase, a biological rotary motor. Trends Biochem. Sci..

[cit9] Nakagawa K., Hirota Y., Sawada N., Yuge N., Watanabe M., Uchino Y. (2010). *et al.*, Identification of UBIAD1 as a novel human menaquinone-4 biosynthetic enzyme. Nature.

[cit10] Kindberg C. G., Suttie J. (1989). Effect of various intakes of phylloquinone on signs of vitamin K deficiency and serum and liver phylloquinone concentrations in the rat. J. Nutr..

[cit11] Wallace B., Young I. (1977). Role of quinones in electron transport to oxygen and nitrate in Escherichia coli. Studies with a ubiA^−^ menA^−^ double quinone mutant. Biochim. Biophys. Acta, Bioenerg..

[cit12] Dowd P., Hershline R., Ham S. W., Naganathan S. (1995). Vitamin K and energy transduction: a base strength amplification mechanism. Science.

[cit13] Fang M., Toogood R. D., Macova A., Ho K., Franzblau S. G., McNeil M. R. (2010). *et al.*, Succinylphosphonate esters are competitive inhibitors of MenD that show active-site discrimination between homologous α-ketoglutarate-decarboxylating enzymes. Biochem.

[cit14] Fang M., Macova A., Hanson K. L., Kos J., Palmer D. R. (2011). Using substrate analogues to probe the kinetic mechanism and active site of *Escherichia coli* MenD. Biochem.

[cit15] Tian Y., Suk D.-H., Cai F., Crich D., Mesecar A. D. (2008). *Bacillus anthracis o*-succinylbenzoyl-CoA synthetase: reaction kinetics and a novel inhibitor mimicking its reaction intermediate. Biochem.

[cit16] Jiang M., Cao Y., Guo Z.-F., Chen M., Chen X., Guo Z. (2007). Menaquinone biosynthesis in Escherichia coli: identification of 2-succinyl-5-enolpyruvyl-6-hydroxy-3-cyclohexene-1-carboxylate as a novel intermediate and re-evaluation of MenD activity. Biochem.

[cit17] Kurosu M., Narayanasamy P., Biswas K., Dhiman R., Crick D. C. (2007). Discovery of 1,4-dihydroxy-2-naphthoate [corrected] prenyltransferase inhibitors: new drug leads for multidrug-resistant Gram-positive pathogens. J. Med. Chem..

[cit18] Basset G. J., Latimer S., Fatihi A., Soubeyrand E., Block A. (2016). Phylloquinone (Vitamin K1): Occurrence, Biosynthesis and Functions. Mini-Rev. Med. Chem..

[cit19] Choi S.-r., Larson M. A., Hinrichs S. H., Bartling A. M., Frandsen J., Narayanasamy P. (2016). Discovery of bicyclic inhibitors against menaquinone biosynthesis. Future Med. Chem..

[cit20] Kurosu M., Crick D. C. (2009). MenA is a promising drug target for developing novel lead molecules to combat *Mycobacterium tuberculosis*. Med Chem..

[cit21] Matarlo J. S., Lu Y., Daryaee F., Daryaee T., Ruzsicska B., Walker S. G. (2016). *et al.*, A Methyl 4-oxo-4-phenylbut-2-enoate with *in vivo* Activity against MRSA that Inhibits MenB in the Bacterial Menaquinone Biosynthesis Pathway. ACS Infect. Dis..

[cit22] Weinstein E. A., Yano T., Li L. S., Avarbock D., Avarbock A., Helm D. (2005). *et al.*, Inhibitors of type II NADH:menaquinone oxidoreductase represent a class of antitubercular drugs. Proc. Natl. Acad. Sci. U. S. A..

[cit23] Lu X., Zhou R., Sharma I., Li X., Kumar G., Swaminathan S. (2012). *et al.*, Stable analogues of OSB-AMP: potent inhibitors of MenE, the o-succinylbenzoate-CoA synthetase from bacterial menaquinone biosynthesis. ChemBioChem.

[cit24] Mathew R. (2010). Inhibition of Mycobacterial Growth by Plumbagin Derivatives. Chem. Biol. Drug Des..

[cit25] Matarlo J. S., Evans C. E., Sharma I., Lavaud L. J., Ngo S. C., Shek R. (2015). *et al.*, Mechanism of MenE inhibition by acyl-adenylate analogues and discovery of novel antibacterial agents. Biochem.

[cit26] Paudel A., Hamamoto H., Panthee S., Sekimizu K. (2016). Menaquinone as a potential target of antibacterial agents. Drug Discoveries Ther..

[cit27] Xu H. , Mechanistic studies of potential drug targets against methicillin-resistant Staphylococcus aureus, State University of New York at Stony Brook, 2009

[cit28] Heide L., Arendt S., Leistner E. (1982). Enzymatic synthesis, characterization, and metabolism of the coenzyme A ester of o-succinylbenzoic acid, an intermediate in menaquinone (vitamin K2) biosynthesis. J. Biol. Chem..

[cit29] Sharma V., Hudspeth M., Meganathan R. (1996). Menaquinone (vitamin K 2) biosynthesis: localization and characterization of the menE gene from *Escherichia coli*. Gene.

[cit30] Lu X., Zhang H., Tonge P. J., Tan D. S. (2008). Mechanism-based inhibitors of MenE, an acyl-CoA synthetase involved in bacterial menaquinone biosynthesis. Bioorg. Med. Chem. Lett..

[cit31] Sharma V., Suvarna K., Meganathan R., Hudspeth M. (1992). Menaquinone (vitamin K2) biosynthesis: nucleotide sequence and expression of the menB gene from *Escherichia coli*. Int J Bacteriol.

[cit32] Truglio J. J., Theis K., Feng Y., Gajda R., Machutta C., Tonge P. J. (2003). *et al.*, Crystal structure of *Mycobacterium tuberculosis* MenB, a key enzyme in vitamin K2 biosynthesis. J. Biol. Chem..

[cit33] Ulaganathan V., Agacan M. F., Buetow L., Tulloch L. B., Hunter W. N. (2007). Structure of *Staphylococcus aureus* 1,4-dihydroxy-2-naphthoyl-CoA synthase (MenB) in complex with acetoacetyl-CoA. Acta Crystallogr., Sect. F: Struct. Biol. Cryst. Commun..

[cit34] Li X., Liu N., Zhang H., Knudson S. E., Slayden R. A., Tonge P. J. (2010). Synthesis and SAR studies of 1,4-benzoxazine MenB inhibitors: Novel antibacterial agents against Mycobacterium tuberculosis. Bioorg. Med. Chem. Lett..

[cit35] Shineberg B., Young I. (1976). Biosynthesis of bacterial menaquinones: the membrane-associated 1,4-dihydroxy-2-naphthoate octaprenyltransferase of *Escherichia coli*. Biochem.

[cit36] Huang H., Levin E. J., Liu S., Bai Y., Lockless S. W., Zhou M. (2014). Structure of a membrane-embedded prenyltransferase homologous to UBIAD1. PLoS Biol..

[cit37] Debnath J., Siricilla S., Wan B., Crick D. C., Lenaerts A. J., Franzblau S. G. (2012). *et al.*, Discovery of selective menaquinone biosynthesis inhibitors against *Mycobacterium tuberculosis*. J Med Chem..

[cit38] Kitagawa W., Tamura T. (2008). A quinoline antibiotic from Rhodococcus erythropolis JCM 6824. J. Antibiot..

[cit39] Debnath J., Siricilla S., Wan B., Crick D. C., Lenaerts A. J., Franzblau S. G. (2012). *et al.*, Discovery of selective menaquinone biosynthesis inhibitors against Mycobacterium tuberculosis. J. Med. Chem..

[cit40] Benkovic S. J., Baker S. J., Alley M., Woo Y.-H., Zhang Y.-K., Akama T. (2005). *et al.*, Identification of borinic esters as inhibitors of bacterial cell growth and bacterial methyltransferases, CcrM and MenH. J. Med. Chem..

[cit41] Hamamoto H., Urai M., Ishii K., Yasukawa J., Paudel A., Murai M. (2015). *et al.*, Lysocin E is a new antibiotic that targets menaquinone in the bacterial membrane. Nat. Chem. Biol..

