# Correction: Menaquinone biosynthesis inhibition: a review of advancements toward a new antibiotic mechanism

**DOI:** 10.1039/c8ra90011f

**Published:** 2018-02-20

**Authors:** M. Boersch, S. Rudrawar, G. Grant, M. Zunk

**Affiliations:** School of Pharmacy and Pharmacology, Griffith University Gold Coast Queensland 4222 Australia m.zunk@griffith.edu.au; Quality Use of Medicines Network, Griffith University Gold Coast Queensland 4222 Australia; Menzies Health Institute Queensland, Griffith University Gold Coast QLD 4222 Australia

## Abstract

Correction for ‘Menaquinone biosynthesis inhibition: a review of advancements toward a new antibiotic mechanism’ by M. Boersch *et al.*, *RSC Adv.*, 2018, **8**, 5099–5105.

The authors regret that there was an error in Fig. 4 in the original manuscript, because the headings for the MIC data were not displayed. The correct figure which includes the headings is shown below. Reference 1 in this correction article refers to reference 37 in the original article.

**Fig. 4:**
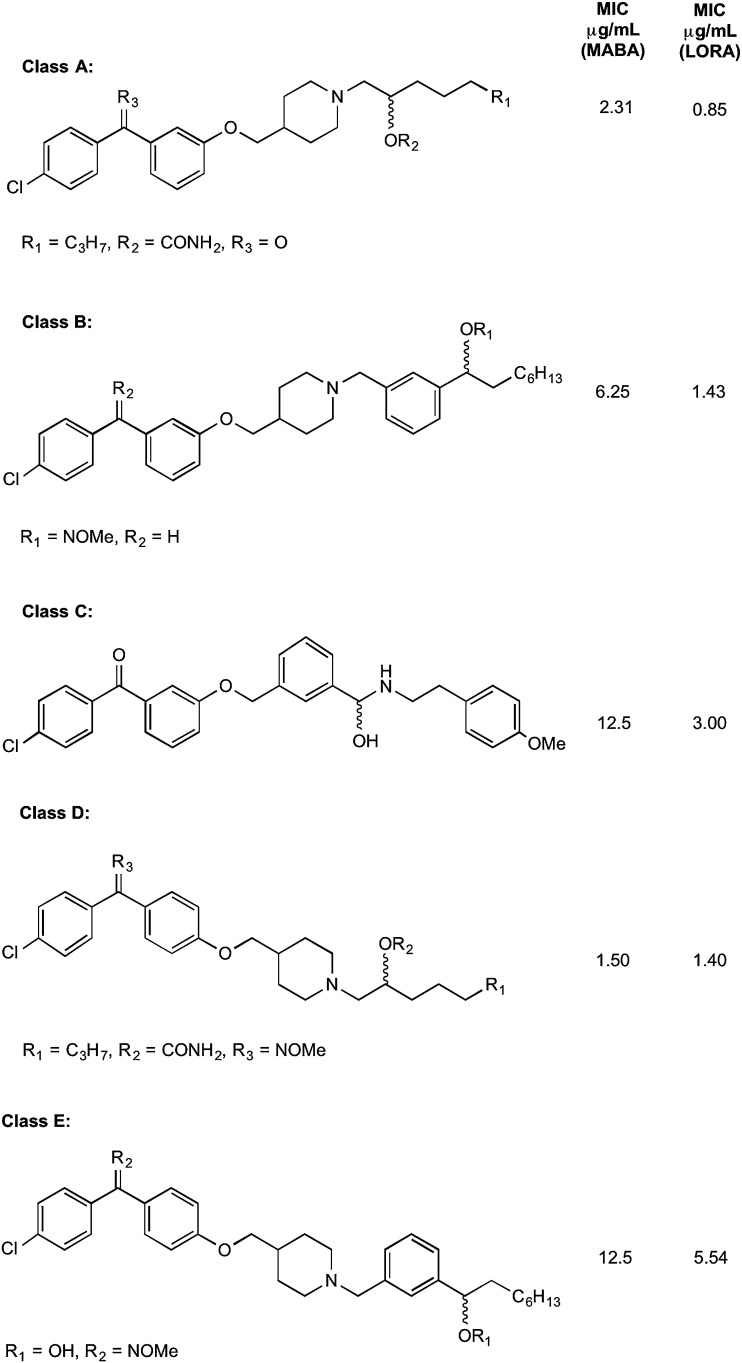
The five classes of compounds discovered by Debnath *et al.* with minimum inhibitory concentrations for both the microplate alamar blue assay, and the low-oxygen recovery assay using *M. tuberculosis*. The MIC values shown are of the best example discovered of each class.^1^

The Royal Society of Chemistry apologises for these errors and any consequent inconvenience to authors and readers.

## Supplementary Material
